# Smart homes, private homes? An empirical study of technology researchers’ perceptions of ethical issues in developing smart-home health technologies

**DOI:** 10.1186/s12910-017-0183-z

**Published:** 2017-04-04

**Authors:** Giles Birchley, Richard Huxtable, Madeleine Murtagh, Ruud ter Meulen, Peter Flach, Rachael Gooberman-Hill

**Affiliations:** 1 Centre for Ethics in Medicine, University of Bristol, Bristol, UK; 2 Policy, Ethics and Life Sciences, Newcastle University, Newcastle, UK; 3 Department of Computer Science, University of Bristol, Bristol, UK; 4 School of Clinical Sciences, University of Bristol, Bristol, UK

**Keywords:** Choice, Engineering ethics, Empirical ethics, Health technology, Privacy, Qualitative research, Research ethics, Smart-home, Ubiquitous computing

## Abstract

**Background:**

Smart-home technologies, comprising environmental sensors, wearables and video are attracting interest in home healthcare delivery. Development of such technology is usually justified on the basis of the technology’s potential to increase the autonomy of people living with long-term conditions. Studies of the ethics of smart-homes raise concerns about privacy, consent, social isolation and equity of access. Few studies have investigated the ethical perspectives of smart-home engineers themselves. By exploring the views of engineering researchers in a large smart-home project, we sought to contribute to dialogue between ethics and the engineering community.

**Methods:**

Either face-to-face or using Skype, we conducted in-depth qualitative interviews with 20 early- and mid-career smart-home researchers from a multi-centre smart-home project, who were asked to describe their own experience and to reflect more broadly about ethical considerations that relate to smart-home design. With participants’ consent, interviews were audio-recorded, transcribed and analysed using a thematic approach.

**Results:**

Two overarching themes emerged: in ‘Privacy’, researchers indicated that they paid close attention to negative consequences of potential unauthorised information sharing in their current work. However, when discussing broader issues in smart-home design beyond the confines of their immediate project, researchers considered physical privacy to a lesser extent, even though physical privacy may manifest in emotive concerns about being watched or monitored. In ‘Choice’, researchers indicated they often saw provision of choice to end-users as a solution to ethical dilemmas. While researchers indicated that choices of end-users may need to be restricted for technological reasons, ethical standpoints that restrict choice were usually assumed and embedded in design.

**Conclusions:**

The tractability of informational privacy may explain the greater attention that is paid to it. However, concerns about physical privacy may reduce acceptability of smart-home technologies to future end-users. While attention to choice suggests links with privacy, this may misidentify the sources of privacy and risk unjustly burdening end-users with problems that they cannot resolve. Separating considerations of choice and privacy may result in more satisfactory treatment of both. Finally, through our engagement with researchers as participants this study demonstrates the relevance of (bio)ethics as a critical partner to smart-home engineering.

**Electronic supplementary material:**

The online version of this article (doi:10.1186/s12910-017-0183-z) contains supplementary material, which is available to authorized users.

## Background

Smart-home technologies—technologies that interact with the home occupier to remotely automate utilities and appliances—exact interest in a range of domains. While the concept of smart-homes has existed since the 1960s [1], the creation of inexpensive wireless devices and powerful computers means that many observers see smart-homes as an idea whose time has come [2, 3]. Interest in smart-homes extends beyond automation for convenience [4] to healthcare applications for older people [5]. A review of technology by Tamura [6] describes using sensors on doors, furniture and appliances, wearable devices and video cameras. Such devices can make physiological measurements, detect activity and inactivity, falls and other accidents. Combined, the information from such devices can track health related routines of smart-home occupants by monitoring their patterns of daily living.

While more studies of smart-homes are focused on the possibilities of data collection [7] than its ethical limits, research has tried to understand the ethical implications of smart-home and related health technologies. These studies commonly raise concerns about privacy, but also suggest consent, social isolation [8] and equity of access [9, 10] are major areas of concern. End-users have been observed to allow their privacy to be compromised if they perceive sufficient personal benefits, which some scholars claim is a fair trade of privacy for increased autonomy [11–13]. Others complain such a compromise neither fosters end-user autonomy nor avoids serious moral harms to personal agency [14, 15]. For example Brown and Adams [9] argue that pervasively observed and monitored individuals will find themselves pressured to alter behaviours in line with particular conceptions of health. While such arguments can appeal to popular notions of personal responsibility, there are good arguments to suggest health and ill-health are normative concepts; [16] what is normal health varies from one time or place to another—consider, for instance, debates about criminal responsibility and mental illness, [17] or changes in attitude toward the status of homosexuality as a health aberration [18]. There are numerous instances germane to smart-homes where provision of a diagnosis brings ethical considerations to the fore. For instance, a diagnosis of dementia raises questions about autonomy, and the suggestion that an accident is a ‘fall’ is a label that may imply frailty and decline. Whether ethical concepts common to bioethics are the most appropriate for discussion of new engineering technologies is the subject of some debate. Distinctive ethical frameworks have arisen in the field of engineering. Floridi has influentially argued that much of bioethical reasoning is “biocentric” and favours an ontological approach to ethics that gives moral consideration to data entities on bases other than whether or not they are biologically alive [19]. His view is not universally embraced, [20] although the need for new forms of ethics that are fit for an age of intelligent machines is widely discussed [21].

These rich dialogues identify numerous ethical issues that may be germane to smart home health technologies, but is largely missing the views of those engineers who work in smart-home research. Orr’s [22] fascinating ethnography of the working lives of photocopier technicians notwithstanding, studies that probe the views and opinions of the engineering community are a relatively slim volume in academic literature, particularly studies of ethical issues [23–25]. Nonetheless their views may be important for a number of reasons. Smart-home researchers play a key role in the inception and implementation of new technologies, and their ethical perspectives may be expected to influence the way ethical problems are responded to. Within a general context this informs a description of the approach of engineering scholars to ethical issues which may directly inform pedagogical or governance perspectives. This task fits within our own empirical bioethics perspective. We are a group of researchers from a variety of academic disciplines, the majority of whom have experience of conducting qualitative studies in order to stimulate (bio-)ethical dialogue about practical situations. Such dialogue may contribute insights useful both to theoretical and practical perspectives. More specifically, these insights might be critically analysed to explore both the problems that are identified and the efficacy of the solutions that are offered to them. While empirical ethics has a rich methodological tradition, [26] the current project takes the relatively modest approach of offering (bio)ethical reflection on the results of a qualitative study we undertook with smart-home engineers. Such an approach, in line with current attempts to foster responsible research and innovation at a European level, [27] aims to contribute to existing dialogue between applied ethicists (including those within our own bioethics community) and the engineering community. The intention is that this will contribute to the ethical rigor of the solutions developed in this area.

## Methods

To investigate the views of smart-home researchers, a qualitative interview study (hereafter “the qualitative study”) was undertaken with researchers on a large project (hereafter “the smart-home project”) concerned with the utilisation of smart-home technologies. To maintain the anonymity of participants we do not identify the smart-home project. It is a large, interdisciplinary research collaboration involving more than 80 academics and support staff at several UK universities including early-, mid- and late-career researchers (respectively defined for this study as PhD students and research associates; lecturers and research fellows; and readers and professors). By examining large sets of data for relationships and patterns (data mining) using multiple data sources (data fusion), the smart-home project aims to address a range of health needs, harnessing existing monitoring technologies that can be deployed in the average home, including depth cameras, wearable devices and environmental sensors. The smart-home project aims to install these technologies in tens of homes, using this experience to create a smart-home health platform that is fit for real world purposes.

Ethical approval to conduct the qualitative study of the researchers and to the publication of anonymised quotations was sought and given by the Faculty Research Ethics Committees at the host Universities.

### Sampling

All early and mid-career researchers in the smart-home project with an engineering role in technology development and deployment were eligible for inclusion, including PhD students, research associates, research fellows and lecturers. The study intended to explore perceptions of social and ethical issues in the smart-home research and we sought to understand the views of researchers who were directly involved in the detail of the design and implementation of smart-home health technologies rather than those who are responsible for the project direction as a whole. We therefore invited early- and mid-career researchers to take part. A list of staff members was provided by the smart-home project administrator; the list gave staff roles, position and contact details. Approaches were made to potential participants in waves of recruitment, with the aim of achieving a sample of researchers who specialised in different technologies. In total thirty researchers were approached, of whom twenty consented to be interviewed.

### Recruitment

Potential participants were approached by email, by letter or in person and given information about the qualitative study. Potential participants were invited to contact the interviewer (GB) if they were interested in being interviewed or if they did not wish to take part. Unless they indicated otherwise, those who did not respond to the initial approach were re-contacted in later waves of recruitment. Recruitment was continued until thematic saturation was reached, understood as the point at which no new themes are judged by the researchers to be arising from the interviews [28].

Potential participants were also familiarised with the qualitative study through a range of activities. The chief investigator (RGH) and the interviewer (GB) attended smart-home project meetings and used them as opportunities to publicise the qualitative study. To ensure that potential participants had the chance to be aware of the researcher and the kind of work he did before deciding whether to not to take part, GB spent time working at a desk in the smart-home project offices. Towards the end of the qualitative study GB gave a seminar to smart-home project researchers about his other work in ethics research, and before the final two interviews he led a workshop with smart-home project researchers exploring some ethical considerations relating to smart-home technologies and public health measures.

### Consent

Potential participants who expressed an interest in being interviewed either met with the interviewer in person or spoke with the interviewer using Skype. The interviewer discussed the qualitative study and what participation would involve with the potential participant and answered any questions. If the potential participant agreed to be interviewed they were asked to sign a consent form, which included consent for the interview to be audio-recorded and consent for extracts from transcriptions of these recordings to be published. Participants were reminded they could withdraw their consent at any time during or after the interview.

### Data collection

Once consent had been provided, a one-to-one, in-depth, semi-structured interview took place. Participants were asked to describe their role and experiences of the smart-home project, and encouraged to offer ethical considerations that they thought germane to broader smart-home design beyond the confines of the immediate project. Areas that seemed pertinent to the qualitative research topic were explored in greater depth. The interview was guided by a topic guide (included in Additional file 1), which indicated major topic areas the interview would explore, and also provided four vignettes, which were designed to facilitate discussion of key topics should they not arise elsewhere in the interview. All participants agreed to have their interview audio-recorded, and these recordings were transcribed by a university approved transcribing service. Transcripts were compared with the interview audio for accuracy and corrections made by the interviewer where necessary.

### Participant characteristics

Twenty participants agreed to be interviewed before thematic saturation was reached. Fifteen of these were men and five were women. Participants were of eight different nationalities including British. Participants included four PhD students, thirteen Research Associates, one Lecturer and two Research Fellows. Seven participants were involved in data mining, data fusion or pattern recognition. The remaining thirteen worked on specific sensors or deployment platforms. Nine participants had spent less than two years working on the project, and the remainder more than two years. Sixteen were from the lead university in the project, with other centres contributing three and one participant(s). Interviews lasted between 52 min and 79 min. Four interviews were conducted by Skype, and the rest were conducted face-to-face.

In publishing these results we are particularly concerned to maximise anonymity of participants. Participant area of expertise is pertinent to the themes, and several quotes are included where this information is given either in the quote or in the discussion. This elevates an already high risk of participant identification within a small research community, even when the usual steps to preserve anonymity, such as pseudonymisation, are taken. Even if widespread identification was impossible, if participants are able to self-identify, this may both undermine the consent that participants have given to have extracts from the interviews published and more generally undermine the confidence of participants in further studies of this type. For this reason, further steps have been taken to increase anonymity; described below.

### Enhancing anonymity

No table of participants is given, as details such as occupation, grade and length of time on the smart-home project would lead to recognition. Given the presence of identifying features in some quotes, simple pseudonyms were found to provide inadequate protection of participants’ identities. However, removal of pseudonyms decreased the readability of the paper and, further, may give inadequate reassurance to readers that quotes from a narrow pool of participants did not dominate the discussion. Nor did removing pseudonyms provide easy traceability between the authors when writing the article. Therefore each participant was assigned two possible pseudonyms, which were securely stored on an encrypted keybreaker table. The cluster ensured that quotes from each participant featured no more than twice in this article, allowed traceability and enhanced readability. Name genders reflected the gender balance of researchers on the smart-home project, but gender was assigned at random to decrease researcher identifiability further. Finally we do not give precise or directly identifying details of the smart-home project in the text of this article, although we are aware that it could be identified by other means. This is primarily because we felt that anonymising the study was a sign of good faith to participants, but also because many discussions in the interviews are about considerations that were broader than the project itself and did not directly reflect the solutions that were adopted within the project.

### Data analysis

Transcripts were analysed using the method of thematic analysis described by Braun and Clarke [29]. Individual transcripts were read and, using NVivo 10, inductive codes were assigned to portions of text by the first author (GB). A total of twenty per cent of transcripts were independently coded by a second member of the study team (RGH, MM, RH, RtM) and codes were checked for consistency. Inconsistent codes were discussed until consensus was reached. When coding was complete, 37 codes had arisen from the text. Similar codes were then grouped into broader themes, which described concepts or understandings that recurred in many interviews. Text that contributed to these themes was re-read in context to ensure the topics reflected the content of the interviews. Quotes that exemplified these themes were selected, lightly edited for readability and participants given pseudonyms.

## Results

Two themes arising from the analysis are discussed here. The first, ‘privacy’, encompassed smart-home project researchers’ discussions about the desirability of personal privacy, the necessity for the privacy of end-users of the technology and the reasons for this. The second theme, ‘choice’ focused on the paradox between solving ethical dilemmas in research by allowing end-users freedom to choose, and the need to ultimately impose ethical judgments that limited end-users’ choices.

### Privacy

Privacy is a “volatile and controversial concept” [30], and is thus difficult to define in a way that satisfies all reasonable objections. Nevertheless, there are clear societal norms protecting personal privacy, even if their boundaries are debated. One taxonomy of invasions of privacy includes:

*the collection, storage, and computerization of information; the dissemination of information about individuals; peeping, following, watching, and photographing individuals; intruding or entering “private” places; eavesdropping, wiretapping, reading of letters; drawing attention to individuals; and forced disclosure of information* [31].

Encouraged to speculate on the problems and possibilities of smart-home technologies, issues germane to privacy arose frequently in the interviews. In particular, smart-home project researchers predominantly characterise privacy as the unbidden sharing of data, rather than as an invasion of personal space.

Researchers vary in their concern about privacy: several indicate they are relaxed about their own privacy. For example, Julian thinks that the loss of privacy is a ‘done deal’, and therefore, not an important worry:

*Julian: if you’ve got a mobile phone, I think people can track you if they really want to. Indeed, I think there are apps where you can, in fact, track your child’s or your partner’s phone, so you do know where they are. Well, that’s “not a big problem”, so I don’t know what is? I’m not too worried about that.*

Similarly, Brian suggests he is relaxed about large-scale commercial collection of personal data:

*Brian: Actually, Google products are really good. If you use them you get a lot of benefit from having - and actually do I really care about what they’re doing with my data? It’s not like that they’re looking at my data personally.*

These responses conceptualise privacy as access to data, and suggest the participants may not contextualise quite distant, person-neutral, corporate information collection as problematic in privacy terms. Even where more personal notes are struck, privacy is often considered in terms of data, rather than in more abstract terms, like being watched. For instance, Margaret and Sally, both researchers who specialise in video technologies, contend that researchers in their field are less sensitive about being filmed in the course of their research than others:

*Margaret: To be honest I think maybe [video researchers] are less sensitive than other people. For video we really want to get that data and we think it is really okay, so if you put it in my home I am okay with it. … I would be okay with [other sensors too].*

Sally offers a similar reflection, which seemed to corroborate this suggestion:

*Sally: I’m a [video researcher] and for years we’ve been videoing each other hopping and walking up and down and I truthfully don’t care if there’s a picture of me doing that in a conference paper at all. There needs to be some balance I think. It’s frustrating as well because I know other research groups don’t do that [gain research approval].*

Although Margaret and Sally’s willingness to share images of themselves in research contexts could, on the face of it, speak of a relaxed approach to being watched, their aims regarding sharing suggest that imaging is again linked to data privacy. Margaret links video to the need for data, while Sally is relaxed about sharing video at a conference of herself moving, as this takes place in a research context. There is no contrast with this data-centred conception of privacy when Sally and Margaret express strong resistance to sharing other types of information about themselves with others: the focus is again on sharing *data*, rather than on sharing prompting more emotive responses (such as feeling exposed). Discussing her resistance to sharing location data, Sally says:

*Sally: A lot of people [share location data] all the time but then you can look at people, you can tell what time they leave work, if they’ve left early, you can see where they go afterwards. I think they’re potty to leave that thing running. … I suppose I just don’t want people knowing exactly where I am at what time of the day. …the journey to and from there and the journey from home to work, I don’t want those patterns of my behaviour being public.*

Similarly, when asked to talk about the types of sensors that might make her uneasy if used outside the project, Margaret expresses concern about sensors that recorded the voice:

*Margaret: For me I probably don’t like voice sensors in there to record the environmental voice in it.*

*Interviewer: So recording speech and so on.*

*Margaret: Yes, the speech things. It is just a personal [thing] for me. I think somehow it is also difficult to process that data. Sometimes people say something and you don’t really want to process that data. Maybe it is better than having the voice recognition system at home or those constrained environment is probably better for users otherwise they have this and they probably think, “Every single word I say will be recorded.” It will always make them think, “That data has to be recorded should I say something?” They always need to think about this so it will probably affect their life.*

Margaret and Sally’s reflections are interesting because their desire not to be located or audio-recorded relates to the unbidden sharing or storage of data. Sharing video is tolerated because it is innocuous data. Location is unacceptable if it is shared with the public. These concerns about data privacy were visible in a range of discussion of specific technologies, including less overtly intrusive smart-home technologies, such as door sensors, noise detectors or, as in the following examples, accelerometers and electricity usage monitors:

*Norman: if someone has access to, let’s say, that data, and he sees you made 2,000 steps, it means if you were home, you’re somewhere else now, because you cannot walk 2,000 steps in your home at a single time.*

*Derek: The reason I don’t like all this data collection is just because I’m a data mining researcher. I know what they can do with the data. … [for example] I can simply use the electricity metering to detect whether someone has played their PlayStation. … [to detect that] this guy is playing video games for the whole night.*

Nevertheless, despite the ubiquity of concerns about keeping data private, some researchers express concerns about privacy at a more visceral level. For example, Gabriel articulates resistance to the idea of deployment of *any* smart-home technology in his own home, stating it would invade his privacy since the home should be a space where

*Gabriel: I can escape from the entire world, and also from the invigilation.*

Such statements are more consistent with a fundamental desire for physical privacy, and this attitude seems to be replicated by other researchers, especially those who express reservations about using the technologies in their own homes:

*Dexter: I don’t want a camera in my house … I don’t like the wearable as well. I don’t like it at all. No, I don’t.*

*Interviewer: For what reason don’t you like it?*

*Dexter: Because, in the home, I just feel like it’s a constraint, too many constraints.*

*Interviewer: Right. So it’s aesthetic for you?*

*Dexter: Yes. I don’t feel comfortable wearing one thing all day. Once you participate you feel that you have to use it.*

Despite Dexter’s agreement with the suggestion that his dislike of a wristband is aesthetic, his final response clearly relies on a notion of physical privacy. There is no suggestion of discomfort at sharing information, the discomfort is more visceral, an invasion of the object into Dexter’s physical space and routines. Similarly Aiden avers cameras are problematic because:

*Aiden: I guess is deeply rooted in the fact that someone has an idea that there is someone on the other side just watching at all times.*

These extracts contain the sort of intuitive expressions of discomfort that may arise from being watched or followed, but privacy is most often discussed in terms of information. When invited to speculate beyond their immediate project, researchers clearly see privacy as a major issue in smart-home research, and are alert to the negative consequences of unauthorised sharing of information. This is unsurprising in one sense, because ensuring informational privacy has the appearance of a tractable problem. On the other hand, physical privacy is much more difficult to address. If potential users of smart-home technologies ‘just feel’ uncomfortable with being monitored by the technology, it is unclear if and how a focus on informational privacy could address the problem. This issue shall be explored further in the discussion section.

### Choice

Privacy is a concept that appears to be linked to notions of consent, so it is unsurprising that ‘choice’ emerges as a second major theme in the interviews. Researchers often appeal to end-user choice to solve the ethical dilemmas inherent in ensuring privacy (and beyond). However a commitment to end-user choice does not sit comfortably with researchers’ roles in defining the boundaries of these choices. Researchers’ discussion of their own choices, which implies certain moral standpoints, and how these might influence—or even constrain—the choices the end-users made, sometimes sharply contradict commitment to end-user choice.

### End-user choices

Researchers frequently invoke provision of choice to end-users in order to circumvent ethically challenging issues, which were in the majority of cases issues germane to privacy. These might be general questions of what data to share; for instance, Angela describes a model where end-users ‘opt in’ to particular smart-home technologies:

*Angela: We provide this, and the end-users should choose about their home, what data they would like to provide. Like GPS is available on my phone, but I can turn it off. When I want some service based on that, I turn it on.*

In a similar way, Frank appeals to the concept of choice to resolve a personal privacy issue raised by the question of whether to store personal data within the end-user’s home or online:

*Frank: [how to solve data storage issues] depends on the people. Some people would prefer to send [data] online and don’t want to see anybody coming into their house and other people would prefer to keep it there and let people in to check it. If people can have a choice, that will be the best plan.*

A further, contentious, issue within the project was the acceptability of recording video images. As we have seen in the first theme collecting video was contentious among some researchers because of privacy concerns, but those in favour argued end-user choice could justify collection of this type of data:

*Elsie: Some users might not even want the camera in their home, so we can’t put the whole thing in their homes. I think that really depends on what the user wants. If they said okay then we are happy to put it in.*

The quotes in this section exemplify a significant sentiment: ethical problems, especially of privacy, could potentially be dissolved if an end-user chose to share their data. Indeed, some researchers take choice to imply responsibility: for example, Oliver suggests the dilemma of gaining consent for (inadvertent) data collection from third parties can be left with the end-user:

*Oliver: If you have third party children coming into the house obviously their guardian would have to say if this is okay or not. It is very difficult. It will probably have to be then on the [end-user]’s shoulders to bear that burden to tell people and inform people. I think it is really difficult.*

On the other hand, some researchers state that personal choice can only go so far. For instance, Godfrey avers that, because a smart-home system could hypothetically indiscriminately monitor anyone who was in that smart-home, the nature of smart-home projects exposes the notion of end-user choice to wider problems:

*Godfrey: [end-users] can say, “I am going to record my own self doing anything I happen to feel like. And I can do that for my own personal ends; whatever those personal ends might happen to me. And that is completely my choice.” And that is legally, if not ethically uncomplicated. The problem becomes when [end-users] have a family and things like that and then you blur the boundaries of what is personal.*

Godfrey indicates that the special problems of dealing with the multiple occupants and visitors, who might include children or non-consenting adults, mean that choice is much more dynamic than simply allowing individual end-users to choose their preference. Firstly, since choices needed to be made *before* the implementation of the system, research choices are potentially a major conduit for researchers’ own value judgements. Secondly, even if there is clear evidence of particular end-user choices, these could not be unrestricted. As detailed in the first theme (‘privacy’), many researchers are concerned that a smart-home environment has hypothetical potential to broadcast data that would damage the end-user if shared in an unrestricted way; as such it is logical that sharing is prevented, even if that prevention brings the choices of end-users and researchers into conflict.

### Making research choices

Researchers voiced near unanimous support for end-user choice to be the arbiter of ethical dilemmas. But researchers also had to make choices about selection, design and implementation of technologies and these by their very nature imply that ethical choices are being made. While these might be framed as choices of a purely technical nature, or choices driven by others, at some stage the researcher themselves has to make basic choices that, plausibly, would affect the ultimate direction of the project. Blake summarises this succinctly:

*Blake: Well, in research, you make your own choices. You decide whether you use this technique or something else, and so on.*

While such an explanation does not explicitly address ethical issues, certain aspects of design clearly took a (very standard) ethical position. For instance, consider this explanation by Gwen of the way the home network was designed:

*Gwen: …if you have a physical network, you don’t really need to encrypt because you just have a cable between device A and device B. Whereas, if you’re using a wireless link, then you definitely have to encrypt data. That basically slows things down, so an advantage of using a wired link is that you can possibly avoid having to encrypt anything. Therefore, you can increase the speed of your storage system.*

Gwen assumes data security—either based on encryption or the inherent features of a closed circuit—to be embedded in design. An ethical choice is made that (for example) there is a duty to end-users to keep data secure. While this is clearly a choice that is bulwarked by societal mores, regulatory norms, the law, and research ethics, it should nevertheless not be overlooked that the opposite choice can be made. It is open to researchers to decide that data is not personally identifiable, and data security can be relaxed. Indeed, it is not uncommon for researchers to report taking such a position, for instance:

*Phoebe: The wristband is basically just accelerometer counts of my hands swinging around as I move around. Maybe it’s got location data that coarsely says which room I’m in in my own home, which is pretty meaningless.*

*Carter: I know for a fact that there’s absolutely, well, nothing they can know about me that I really don’t want them to know. The microphone is not able to record sound or send a sound anywhere else. The accelerometer data is literally just accelerometer data and at the end of the day there’s really only so much you can find out from that. You can’t really find out if someone is doing any specified task.*

Yet, while researchers tend to defer to research guidelines in spite of such value judgements, some ethical questions required a decision to be taken at the design stage. For instance, should the project focus on technologies with a high risk of personal identifiability (like video)? Where is the balance between gathering valuable data and user acceptability? What is practical? What is relevant? Some of these questions about what should be monitored had been put to clinicians, but Florence highlights the ultimate need for researchers to discover what information to gather:

*Interviewer: Presumably you’re collecting this data because clinicians have said that this type of data would be useful?*

*Florence: Yes. That’s a question for us, as well. In the very beginning, we didn’t know, even though we had a literature review. Many literature said that’s a wearable sensor, so you can use it to detect that kind of activity. Daily activity. But that’s not a strong conclusion to say that we need these sensors, these sensors, and that this list of sensors can give you this list of activity recognition.*

*Interviewer: So you’re almost collecting data to see what you can find out.*

*Florence: That’s one of the tasks we need to do. We deploy a list of sensors, to see which one, what list of sensors is actually useful to detect the daily living activity of the user.*

Florence’s comments point toward a paradox of experimental research. To decide what to monitor, the researcher must first start monitoring. While discussions with clinicians and the public could be useful to guide these decisions, the researchers explain that some guesswork was always needed. For example, Connor indicates the need for researchers to anticipate what end-users would accept:

*Connor: Basically, we are basing our choices on the fact that we think that the [end-users] that volunteer are happy to do that.*

Connor’s comment contains an echo of the earlier appeal to end-user choice. Indeed, design choices are not framed as explicitly restrictive of ethical choices, but simply technical decisions. Nonetheless, as stated earlier, allowing end-users to follow their preferences in unrestricted ways is ultimately problematic for some researchers, and leads to discussion of controls on end-user behaviour, although such controls are rarely, if ever, made explicit to end-users.

### Influencing end-user choice

In some instances, researchers describe decisions that had been explicitly made to prevent end-users from making ‘bad’ choices. Sometimes these were framed, as above, as technical decisions, but these technical considerations merge with clear intentions to prevent users from acting in certain ways. At a whole-project level the smart-home project would not store ‘raw’ data as collected by sensors. Instead, such data would be processed immediately by the system (a server) within the home into higher-level data, and the system in the home would not be connected to the wider internet beyond the home. In the following exchange, Seth justifies restrictions to end-user access to processed data, because access may cause them to reflect negatively on their actions:

*Seth: [end-users] shouldn’t have access [to their data]. Because when you look at your own data, you feel like you didn’t do very well.*

Other researchers suggest that end-user data should be shared with health professionals, but that the decision about what to share should rest with researchers, rather than the end-user themselves:

*Austin: But I think the user isn’t necessarily to be the one to decide what data they will share.*

As with Mia’s statements about allowing users to record video, such questions could still conceivably be framed as technical choices, so rightly in the ambit of researchers to make. However several researchers assert that end-users may use the technology for ethically suspect purposes. Roger is among several researchers who found the possibility that parents could hypothetically use smart-home technology to monitor their children’s behaviour ethically troubling:

*Roger: [a smart-home system] would really have to be well thought out to avoid misuse … let’s say that you’re living with your children in such a house that you can monitor their every step. I think that’s not appropriate, or that’s not ethical. You would not want your children to behave good because you’re monitoring them, but you want them to behave in a good way because that’s the right thing to do.*

These sentiments are clearly at odds with previously noted sentiments that proposed end-user choice—and the wider principles of end-user consent—as a justification for the collection and sharing of personal data. Yet, allowing end-user choice remains important for most researchers. For one thing, researchers perceive difficulties in preventing end-users from using the system as they choose. As Leon says:

*Leon: I think [poor end-user choices are] the concern of the project, yes. I think it’s very difficult to force people. Not only that it might be ethically questionable, but I think it’s technically difficult to force them not to use the system in this way, or that way.*

Thus, with regard to the dilemma of monitoring their own children, Elliot states that end-users, armed with the correct information, would make the right choice about their monitoring their children:

*Elliot: I mean, we should still want these people to choose what the right thing is for them on their own free will.*

*Interviewer: Right, so you want people to make the right choice?*

*Elliot: We should give the information so they are able to make the right choice.*

Elliot’s appeal, framed using the language of information, choice and free-will, closely traces the conceptual language of consent, and emphasises researchers’ reluctance to impose choices overtly on end-users, even where a need to minimise end-user’s choices to ethically acceptable options is asserted.

Choice was a major area of discussion in the interviews. Many researchers suggest that end-user choice might solve ethical dilemmas; indeed, given the preponderance of researchers favouring end-user choice as a way to solve ethical dilemmas, this may suggest a libertarian slant toward their thinking. Nevertheless researchers also indicate that end-user choices can be restricted. Such restriction is almost always done covertly, through design, rather than in ways that might overtly challenge the notion of end-user choice. The values of researchers might be channelled into the basic design choices they make, but the ethical dimension of design was rarely acknowledged. The implications of this and the previous theme are discussed below.

## Discussion

The views and experiences of smart-home researchers indicate that privacy and choice are their major areas of ethical concern. Their approaches can be analysed by asking two major ethical questions, which we invite smart-home researchers to reflect upon:

1. Researchers identify privacy as a major concern in the conduct of research, yet tend to see privacy in terms of informational privacy, and only rarely see privacy as physical privacy. Is this focus on data privacy appropriate?

Although some form of respect for privacy is a global societal norm, whether and where privacy can coherently be termed a moral concept is deeply contested [32], since respect for privacy explicitly places restrictions on enforcing other moral claims. Perhaps because of this contested moral value, moral claims based on privacy are at the heart of many polarised bioethical debates [30]. Philosophical debates about privacy centre on whether privacy is an interest in its own right (and thus is vulnerable to harms), or instead a cluster of other more basic interests, such as property interests and liberty interests [33]. Privacy is clearly addressed in law; in England and Wales, the Article 8 right to respect for privacy and family life of the Human Rights Act 1998, the Data Protection Act 1998 and the Regulation of Investigatory Powers Act 2000 are important contemporary statutory examples. Legal discussion that dates back to the 19^th^ century [34] indicates the longstanding (though sometimes problematic) place of privacy in culture.

While various typologies of privacy exist, the way that privacy was discussed by smart-home researchers can be seen to follow a division of privacy into ‘physical privacy’—avoiding unwanted sensory access—and ‘informational privacy’—avoiding the misuse of information [35, 36]. In support of such a division, NA Moreham [36] makes the plausible claim that watching or following a person breaches their privacy even if no information is obtained or circulated. Setting aside the philosophical debates that this claim invites, there is a clear practical implication for those working in this field. Viewing researchers’ discussions of privacy through the lens of informational and physical privacy gives insight into the challenge of developing and delivering home monitoring technology to members of the public. Maintaining informational privacy may appear to be a tractable challenge, given the advanced state of technologies such as encryption. Moreover, by isolating networks from the internet, basing servers in the home and so forth, the researchers had made considerable efforts to reduce the likelihood of unauthorised data sharing. While there is always some possibility of unanticipated data breaches, researchers were concerned to identify and avoid such possibilities.

Concerns about physical privacy appear to be less tractable than concerns about informational privacy. These concerns therefore pose perhaps a greater threat to the uptake of smart-home technologies, and, in the short term, to delivering smart-home technology to users. It is difficult to gauge the scale of this problem. The positive levels of user acceptance of sensing technologies seen in some studies [37] have limited value due to their small scale. Research has also tended to focus on people living with long-term conditions [38], who may react more positively than those less invested in monitoring their health; by contrast, a third of users of wearable health technologies stop using these products within six months [39]. Moreover, research has focused on discrete technologies [40, 41] rather than the ubiquitous sensors and monitoring devices envisaged as the end-point of smart-homes. End-users’ responses to these technologies in the field have much to tell us. Some studies of end-users suggest that unobtrusiveness and invisibility are valued at least as highly as assurances of informational privacy [42]. Such tendencies toward physical privacy may manifest in the rejection of some or all types of smart-home monitoring that cannot be overcome by reassurances about data protection. Quite how these issues can be solved in the context of smart-homes ultimately demands input both from the people who use their technologies and those who actively reject such technologies, about how they feel. Until this is investigated, we should be wary of overestimating what can be achieved with data privacy.

2. Researchers saw offering end-user choice as an effective way of addressing ethically sensitive issues, but also acknowledged end-user choices should be restricted in some instances. How much choice should end-users be offered?

Writers often link choice to autonomy, and both healthcare ethics [43, 44] and the ethics of research participation are concerned with patient or participant choice. Some bioethicists reduce most ethical dilemmas to respect for autonomy [43]. They justify this position by referring to a plurality of values and the lack of an overarching moral consensus. However, this position is often guided by libertarian motives and extreme emphasis on individual freedom [45]. In this case, they might argue that, if individual can agree with the intrusion of their life sphere, there is no ethical issue any more. In our study, researchers’ inclination to offer choice to future end-users could be interpreted in two ways. First, as described in the results section, reducing ethical dilemmas to choice could allow smart-home researchers to avoid taking responsibility for ethical decisions. Second, the desire to offer choice may relate to a libertarian approach to end-user choice in which choice is seen as a completely individual process in which no others are (or should be) involved. The first of these is more pragmatic in orientation and the second is a more principled basis for provision of choice. Both possibilities may operate alongside each other, or in an intertwined manner, which accords with research in other contexts. For instance, Mol observed that the rhetoric of autonomous choice in healthcare can be interpreted both as rationale by practitioners to excuse themselves from caring about the patient who makes choices *and* as principled (we would say libertarian) respect for those patient choices [46].

The nature of privacy is “one of the most obvious and pressing issues in computer ethics” [47] and a vexed one in ubiquitous computing [48]. If the researchers in this study largely endorse the thesis that choice can resolve ethical issues (whether for principled or self-serving reasons), it is germane to ask how choice might relate to personal privacy. The close identification of privacy with choice is only reflected in some areas of existing literature, mainly in context of U.S. constitutional law and related academic literature, where privacy has been argued to underpin rights to autonomy [49]. However, many writers have not taken this approach, [50] for instance, Nissenbaum [32] argues that personal privacy is measured by degree of access rather than degree of choice. Similarly, Cate suggests, “individual choice is not the same as personal privacy, so focusing laws, regulations and enforcement on the former will not necessarily enhance the latter.” [51]. Cate also argues that a focus on choice allows data collectors to shirk their obligations to maintain privacy, shifting responsibilities in relation to privacy onto the individuals who provide the data. This analysis suggests that an inappropriate focus on choice may also raise social justice concerns. Furthermore, asking end-users to make frequent choices about participation (which we may wish to do in smart homes, with their perpetual monitoring) may place excessive burden on individuals [51, 52]. Other contexts where patients and consumers are routinely presented with choices, such as informed consent documents, terms of credit documents or software terms-of-use contracts have been empirically demonstrated to result in poor exchange and understanding of information [53]. Therefore, offering choice may not be the solution to privacy issues, may place burden on individuals, may not enhance understanding and may raise social justice concerns.

The imperfections in ensuring privacy by providing more choice may be behind the tensions about the role and extent of choice within the interviews. By disconnecting choice from personal privacy we might begin to explore what other benefits and harms flow from choice (a number of which were identified by researchers). Possible benefits may include a feeling of reassurance to end-users that a smart-home system is delivered in a professional manner and the sense that they are able to withdraw if use of the system is burdensome. Possible harms that should be guarded against include giving end-users the ability to monitor others within smart-homes without appropriate consent, disenfranchisement if end-users are given information that they do not understand, and negative impact on end-users if they are provided with access to data about themselves without interpretation and support. These harms could be serious, and may be justifiable reasons for restriction of some choices to end-users. Such restrictions should be monitored to ensure appropriateness and carefully balanced against possible benefit of broader choices, informed by ethical reflection on the meaning of privacy as a widely shared value. By decoupling choice from privacy, the duty to protect the privacy of end-users and third parties is placed more firmly with researchers.

### Limitations

The study has some limitations. The lead researcher was not a smart-home engineer and thus may have interpreted technical distinctions in a naïve way. However, this naïvety is generally considered an advantage in qualitative research, since the researcher investigates basic assumptions. The participants were early- and mid- career researchers and so the material presented here accurately reflects their views rather than decisions about the smart-home project as a whole, which are not explored within this study. However, the study is intended to reflect on smart-home research in general rather than on particular solutions adopted in a single project. The study only investigates the views of the researchers and not of the end-users, and it will be important to gather the views of end-users to be able to compare the views of users and engineers. We recruited from a single smart-home project, and it is possible that the micro-culture does not reflect experiences in other projects. As study participation was voluntary it is possible that the researchers who took part had particularly keen or specific interest in the topics that the study addressed. Despite this we have confidence in the findings because we encountered diverse opinions and were able to include participants from several institutions. We appreciate that interviewing and thematic analysis are subjective processes, and it is possible that other researchers would have encountered different themes. However, we conducted interviews and analysis until thematic saturation was achieved, and the process of analysis included double coding and deliberative discussions within our experienced multi-disciplinary research group. We therefore suggest that the interpretation presented here is appropriate and germane to the developing research in this area.

## Conclusions

Applying smart-home technologies to healthcare applications represents not only a technical, but an ethical, challenge. Ethical analyses of these technologies raise concerns *inter alia* about loss of privacy, the adequacy of consent and maintaining agency. Exploring the views and experiences of researchers of smart-home technologies brings insights into which ethical issues researchers see as pertinent to their technologies, and the approaches that they take to solve these issues.

Qualitative interviews with researchers in this large, multi-centre smart-home project indicated that privacy and choice were the major areas of ethical focus in the design and implementation of smart-home health technologies. Researchers primarily viewed privacy as a problem of data security, and expended efforts to develop a system that would minimise the exposure of data to the outside world. Laudable as such an approach is, a focus on data privacy may take too little account of the much less tractable problem of ensuring end-users felt that their physical privacy was respected. While actual respect is clearly ethically important, favourable end-user perceptions are essential to public acceptability of new technologies and thus ensuring that their benefits are spread equitably. Even where researchers were able to ensure adequate data privacy, the lack of a commonly agreed concept of privacy could mean that, even with sustained attention, privacy is limited in its solubility as an ethical problem. Essential to developing approaches to these problems will be to continue research with those end-users who both engage, and do not engage, with smart home technologies.

Researchers also indicated considerable efforts in ensuring end-user choice were addressed in the system design. Many saw this as a ready solution that addresses many of the ethical problems involved in maintaining privacy. At the same time, the interviews revealed awareness among researchers of the limitations of this strategy, and despite an overwhelming endorsement of the moral desirability of user choice many researchers discussed elements of design which restricted such choice. Researchers tended to describe these as technical solutions to engineering problems, and did not seem to explore or question the normative assumptions that accompanied them. Researchers overtly discussed restriction of end-user choice in only a few cases and even then, with extensive caveats. This may point to a desire to avoid making ethical choices, but might equally be consistent with a pro-autonomy position. Choice may then, be a way to address ethical issues. Nevertheless, given the pervasiveness of privacy concerns in ubiquitous computing, it is notable that identification of privacy with choice is not a ethically acceptable strategy. Indeed some privacy scholars suggest it misidentifies the sources of privacy and risks the unjust burdening of end-users with a problem that researchers themselves have greater resources to address. Such analysis suggests that considering choice and privacy as separate issues is likely to result in a more satisfactory treatment of both.

Our research aimed to document the perspectives of researchers in a rapidly developing technological field. We intend, through this engagement, to demonstrate that (bio)ethics can be a critical partner to smart-home engineering. We suggest that some of the possible ‘critical’ roles of the ethicist in such a partnership could be to identify the moral issues, to help to resolve them, to point at issues that are intractable or difficult to resolve and to find ethical justifications or express concerns. Such collaboration between ethics and engineering is necessary to develop solutions that are both technologically and ethically robust in an area that is likely to be of increasing importance in healthcare delivery.

## Additional file

Additional file 1: Smart homes private homes: interview topic guide. (DOCX 20 kb)

## Abbreviation

U. S: United States
