# Supplementary material for: Smart homes, private homes? An empirical study of technology researchers’ perceptions of ethical issues in developing smart-home health technologies
Source: BMC Med Ethics. 2017 Apr 4;18:23. doi: 10.1186/s12910-017-0183-z (PMC5379767; doi:10.1186/s12910-017-0183-z)
Supplement: Additional file 1: — Smart homes private homes: interview topic guide. (DOCX 20 kb) [file 12910_2017_183_MOESM1_ESM.docx]

Interview topic guide

**Before interview starts: Researcher will discuss the study with the potential participant, prior to the dialogue described here, the information sheet will be given to the participant and the contents discussed. Participants will be given the opportunity to ask any further questions before they are asked if they would consent to the interview taking place. Participants will sign a consent form, and a copy will be sent to them afterwards. The audio recorder will be turned on.**

**This is not intended to be a precise script. The following questions and dialogue are illustrative of the kind of questions which will be asked at interview, and should be used flexibly.**

**Part 1: about you and about role/experience of [smart home project]**

- Age, gender, occupation
- What sort of technology do you use in your free time?
  - Where do you use this technology?
  - How would you describe your relationship with this technology?
  - Who has sight of the data
- Current role/experience. How long in this role/or known about [smart home project]

**Part 2: about experience of data in [smart home project]**

- What kind of data do you deal with in [smart home project]?
- How is it collected? (sensor type)
- How is it stored? (where, format, anonymity)
- How is data transferred (from house, and where to?)
- Who uses the data, and what for?

**Part 3: about wider issues relating to storage and sharing of data**

- If you were living in a home with the sensors installed, or, if your home had the sensors installed, what things would you want them to collect?
  - What things would you would not want them to collect?
- To explore these issues further, I’d like to discuss three examples of potential problems with misuse of shared data. In each case I would like you to consider how this affects your thinking about the types of data you would be (un)comfortable being collected.

**The participant will be given a written copy of a vignette that will be read by the researcher. This section contains four vignettes, one or more of which will be presented to participants if pertinent issues are not raised in elsewhere in the interview.**

**Vignettes will both seek inductive themes arising from participants thoughts about [smart home project] technology, as well as deductive themes that ask participants to reflect on problems that are commonly claimed to relate to data storage and sharing.**

**Inductive vignette 1:**

You have the chance to try out a new wearable wristband sensor for a week. It looks a bit like a watch. You are keen to do this as you want to know about how long the battery life is, what it is like to wear one and what you can find out about yourself.

- What data would you like it to collect? Why?
- What data would you not like it to collect? Why?
- What would you like to happen to that data? Why?
- Who should see that data? Why?

**Inductive vignette 2:**

You are working as a staff member in a project that is collecting data from a wearable wristband sensor. You have the chance to decide what data is can collect and what happens to the data. Imagine that there are no practical limitations on this.

- What data would you like it to collect? Why?
- What data would you not like it to collect? Why?
- What would you like to happen to that data? Why?
- Who should see that data? Why?

**Deductive vignette 1:** International data storage

Personal health data from the wristband is managed by a European company who extract health information from the data and upload it in bulk to a server in a third country. While the server remains safe (the business provides valuable income for the local economy), the province where the server resides has recently been taken over by rebels loyal to a hostile neighbouring nation.

- What do you think about this situation?
- What sort of concerns if any, would it raise?
- Would there be any types of data you would consider particularly sensitive?

**Deductive vignette 2:** Commercial use

Data from the wristband is shared with your GP. Recent changes in the NHS have meant that the management of this data is outsourced to a for-profit telehealth company. The company have close links to a large USA-based health insurer. Since the changes, you have started receiving telephone calls from this health insurer offering you tailor-made private healthcare packages. The salesperson is very persuasive, and explains that the data he has about your health indicates you could get some very competitive deals on their latest products.

- What do you think about this situation?
- What sort of concerns if any, would it raise?
- Would there be any types of data you would consider particularly sensitive?

**Part 4: Close**

- Anything you want to say that you have not yet said?
- Any questions for me?
- Would you like to receive a leaflet containing a summary of the findings of the study?

**At end of interview**: thank the participant and provide a copy of their signed consent form, either immediately (if a copier nearby) or by post later.
